# Editorial Notes

**Published:** 1923-01

**Authors:** 


### Editorial Notes.

**Vol. XL.**

AFTER "seven lean years," 1915 to 1922, with this issue the fortieth volume of our *Journal* enters a new year, and we trust a new era, for we are resuming our pre-war plan of publishing four quarterly numbers. We have been through troublous times, for during the Great War the meetings of the Society were relatively few, most of our members being either abroad on active service or deeply involved in military duties or taking work for colleagues so engaged. The Armistice and the days of declared peace did not end our difficulties, as the immensely increased cost of printing and even of paper placed insuperable obstacles in the way of producing the *Journal* on the old-time scale, the Society being, therefore, compelled to restrict the size and number of its issues. Nevertheless, we have cause to be thankful that we have survived vicissitudes which all Medical Journals have suffered from and in some cases succumbed to, and for this we are indebted to the support afforded by the Society and in no small measure to the helpful co-operation of our publishers and printers.

Our first Quarterly number appearing in January, we are thus enabled to begin the volume with the Presidential Address, and the succeeding issues will be published in March, June and October.

**Boylston Medical Prize of Harvard University.**

THE Boylston Medical Committee of Harvard University announces that for 1922 there is offered a prize of five hundred dollars and the Boylston Prize Medal for the best dissertation on the results of original research in medicine, the subject to be chosen by the writer. The Boylston

Prize Medal will be added to the money prize only in case the winning essay shows special originality in the investigations detailed.

These prizes are *open to public competition,* and in awarding them preference will be given to dissertations which exhibit original work, but if no dissertation is considered worthy of a prize the award may be withheld.

Each dissertation must bear, in place of the author's name, some sentence or device, and must be accompanied by a sealed packet, bearing the same sentence or device, and containing the author's name and residence within.

*Any clue by which the authorship of a dissertation is made known to the Committee will debar such dissertation from competition.*

Dissertations must be printed or typewritten, and their pages must be bound in book form.

A similar announcement will be made for 1923.

Dissertations entered for this prize must be in the hands of the Secretary, Reid Hunt, M.D., Harvard Medical School, Boston, Mass., on or before February 1st, 1923.

---

